# Adaptively Dense Feature Pyramid Network for Object Detection

**DOI:** 10.1109/access.2019.2922511

**Published:** 2019-06-12

**Authors:** HAODONG PAN, GUANGFENG CHEN, JUE JIANG

**Affiliations:** 1 College of Mechanical Engineering, Donghua University, Shanghai 201620, China; 2 Department of Medical Physics, Memorial Sloan Kettering Cancer Center, New York, NY 10044, USA

**Keywords:** SSD, object detection, atrous convolution, DenseNet, SENet

## Abstract

We propose a novel one-stage object detection network, called adaptively dense feature pyramid network (ADFPNet), to detect objects cross various scales. The proposed network is developed on single shot multibox detector (SSD) framework with a new proposed ADFP module, which is consisted of two components: a dense multi scales and receptive fields block (DMSRB) and an adaptively feature calibration block (AFCB). Specifically, DMSRB block extracts rich semantic information in a dense way through atrous convolutions with different atrous rates to extract dense features in multi scales and receptive fields; the AFCB block calibrate the dense features to retain features contributing more and depress features contributing less. The extensive experiments have been conducted on VOC 2007, VOC 2012, and MS COCO dataset to evaluate our method. In particular, we achieve the new state of the art accuracy with the mAP of 82.5 on VOC 2007 test set and the mAP of 36.4 on COCO test-dev set using a simple VGG-16 backbone. When testing with a lower resolution (300 × 300), we achieve an mAP of 81.1 on VOC 2007 test set with an FPS of 62.5 on an NVIDIA 1080ti GPU, which meets the requirement for real-time detection.

## I. INTRODUCTION

In recent years, deep convolutional neural networks (CNNs) have fostered the development tasks in computer vision, such as classification [1]–[4], semantic segmentation [5]–[7] and object detection [8]–[11] through learning better feature representations. For example, to extract high level information, VGG [1] uses very small (3 × 3) convolution to deepen the network. For the same goal, GoogLeNet [2] proposes an inception module to increase the depth and width of the network. The introduction of shortcut by ResNet [3] makes the backward propagation of the gradient easier and enables the deeper networks to be effectively trained. DenseNet [4] connects every layers in a feed-forward approach to strengthen feature transmission, encourage feature reuse, and improve feature expression.

As for the object detection, the purpose is not only to identify the class of objects, but also to localize the object within a bounding box. At present, CNN features have better robustness and strong characterization ability than traditional hand-crafted features. Traditional image processing, which is characterized by hand-crafted features, relieves the problem of multiple sizes of objects by constructing an image pyramid [12]. The pyramid of an image is a set of images that are progressively reduced in resolution and derived from the same original image. Due to its effect in analyzing images at different scales, the image pyramid is also introduced into deep CNNs based object detectors.

Nevertheless, images in the pyramid need to calculate features separately, which greatly consumes computing resources. Therefore, Single Shot MultiBox Detector (SSD) [13] designs a pyramidal feature hierarchy to reuse the multi-scale feature maps and detects objects of different sizes at each feature layer. This method greatly reduces the waste of resources compared to the image pyramid. However, SSD uses features from shallow to deep, resulting in high-resolution feature maps without sufficient semantic information. Furthermore, Feature Pyramid Network (FPN) [14] constructs a top-down framework with lateral connection to produce feature maps with strong semantic information at all scales.

Recently, it has been shown that receptive field plays a key role in object detection in various scales [15]–[17]. For example, inspired by the Atrous Spatial Pyramid Pooling (ASPP) [18], aggregating features through series atrous convolutions with different atrous rates is introduced in [15], [16] for object detection. Unlike increasing the field of view through traditional convolution, the atrous convolution alleviates the contradiction between the field of view and feature resolution, which might be benefit for object localization and detection.

In order to better promote the development of multi-scale object detection, we propose a novel network structure, named adaptively dense feature pyramid (ADFP), which enhances the feature representation capabilities of CNNs-based network structures. The network structure is mainly composed of dense multi scales and receptive fields block and adaptively feature calibration block. The dense multi scales and receptive fields block mainly consists of a cascade of atrous convolution layers through densely connection, resulting dense multi-scale features from multiple receptive fields. Then the adaptively feature calibration block is used to calibrate the produced feature maps based on the feature dependencies to retain features contributing more for the detection and depress features contributing less. We then construct a novel one-stage object detector based on SSD [13] framework. By introducing structure designed by us, the new object detector not only achieves state-of-the-art performance, but also maintains faster detection speed. Our work is most related to the work in [15]. While the difference lines in that we use dense connections to extract features and the features is calibrated by a followed SENet module. Our experiment results confirmed our approach by keeping high level semantic information and fine details simultaneously for a object detection task. In summary, the contributions of the this paper are listed as follows:

1. A novel module called adaptively dense feature pyramid (ADFP) to densely aggregate information at multi scales and receptive fields is proposed.
2. The proposed ADFP module is seamlessly applied in the framework of SSD constructing a novel one-stage object detector that has achieved significant accuracy improvement as well as rapid detection speed.
3. We conduct extensive experiments on the public benchmarks of Pascal VOC and MS COCO to evaluate our detectors. Compared to other VGG-based detectors, we achieve new state-of-the-art results without any bells and whistles, which demonstrates the effectiveness of our proposed module.

## II. RELATED WORKS

### A. OBJECT DETECTION

In the recent years, deep convolutional neural networks (CNNs) have promoted the progress of object detection. Compared to traditional hand-crafted features, such as HOG [19] and SIFT [20], CNNs are able to learn better feature representation. In general, those CNNs based frameworks can be roughly grouped into two categories: two-stage detectors and one-stage detectors.

#### 1) THE TWO-STAGE DETECTORS

The two-stage detectors construct a classical framework, where the first phase generates a series of object proposals and the second phase further refines proposals’ bounding boxes and classifies the proposals with the features produced by CNNs. The R-CNN [21], which firstly introduces the CNNs into the task of object detection, adopts the Selective Search [22] to produce a sparse set of region candidates and refines them through a CNNs model. SPPNet [23] eliminates the limitation of image size ratio by introducing the spatial pyramid pooling strategy. To overcome the weaknesses of R-CNN and SPPNet, Fast R-CNN [24] implements end-to-end training, which can update all network layers. In order to further speed up the detection, Faster R-CNN [25] introduces a Region Proposal Network (RPN), which enables nearly cost-free region proposals. The RPN shares the features from backbone with the detection network and is trained end-to-end to simultaneously predict object bound boxes and class scores on region proposals regions. R-FCN [26] achieves a fully convolutional network for object detection by position-sensitive score maps. Aiming to the objects at different scales, FPN [14] constructs a top-down horizontal connectivity architecture, which combines the strong semantic feature and the high resolution feature to rebuild a fully semantic feature pyramid. By adding a branch of predicting masks on Region of Interest (RoI) to the Faster R-CNN, the Mask R-CNN [27] implements both instance segmentation and object detection simultaneously. Although the two-stage detectors have shown dominated performance, their two steps is computationally intensive for fast detection.

#### 2) THE ONE-STAGE DETECTORS

To accelerate the lower detection speed in two-stage detectors, some researchers divert attention to the one-stage detectors to balance the accuracy and speed. Without the proposal generation step, the one-stage detectors directly predict confidences and locations from full images in a single network end-to-end. For example, with a multi-scale and sliding window, OverFeat [28] applies a classification-trained network to predict object bounding boxes and classification. You Look Only Once (YOLO) [29] treats object detection as a regression problem, using a single network to directly predict bounding boxes and class probabilities. Although there is a slightly lack of precision, YOLO is extremely fast. After that, YOLOv2 [30] and YOLOv3 [31] are proposed to further improve the accuracy in various aspects. Among those one-stage detectors, SSD [13] detects objects of a certain scale through a series of pre-defined anchor boxes on corresponding layers. The anchor boxes over different aspect ratios and scales are set on each layer of a feature pyramid. DSSD [32] replaces the backbone with Residual-101 and adds a large number of high-level semantic information by deconvolution to improve the accuracy. To inherit the merits of both one-stage detectors and two-stage detectors, Single-Shot Refinement Neural Network (RefineDet) [33] designs two inter-connected modules, the anchor refinement module and the object detection module, to coarsely refine the anchors and further improve the regression and classfication respectively. Kong *et al.* [34] reformulate the feature pyramid structure to combine deeper features and shallower features with global attention and local reconfigurations. To enrich the semantic information for features, Zhang *et al.* introduce a semantic segmentation branch and a global activation to build Detection with Enriched Semantics (DES) [35]. Parallel feature pyramid network (PFPNet) [17] increases the width of the network instead of deepening the depth of the network to avoid integration between different layer features.

### B. ATROUS CONVOLUTION

The traditional CNNs increase semantic information or receptive fields through a series of convolutional filters as well as pooling layers. However, it reduces the image feature resolution, which is important for accurate object localization and detection. To alleviate the contradiction between sufficient receptive field and image feature resolution, atrous convolution [36] or dilated convolution, is proposed for the task of semantic image segmentation and later developed by [18], [37]–[39] for semantic segmentation. Recently, as in the field of object detection, Liu *et al.* [15] build a lightweight model based detectors, Receptive Field Block Net (RFBNet), using a RF Block (RFB) module to enhance the feature discriminability and robustness. As opposite to this method using atrous convolution, we propose to use a dense connection of atrous convolution, which produces features densely in both scales and receptive fields. Those generated dense features are then calibrated by a adaptively feature calibarion block to retain features contributing most for the detection task and depress features contributing less. The details of our proposed module is described in the following section.

## III. METHOD

**FIGURE 1.**
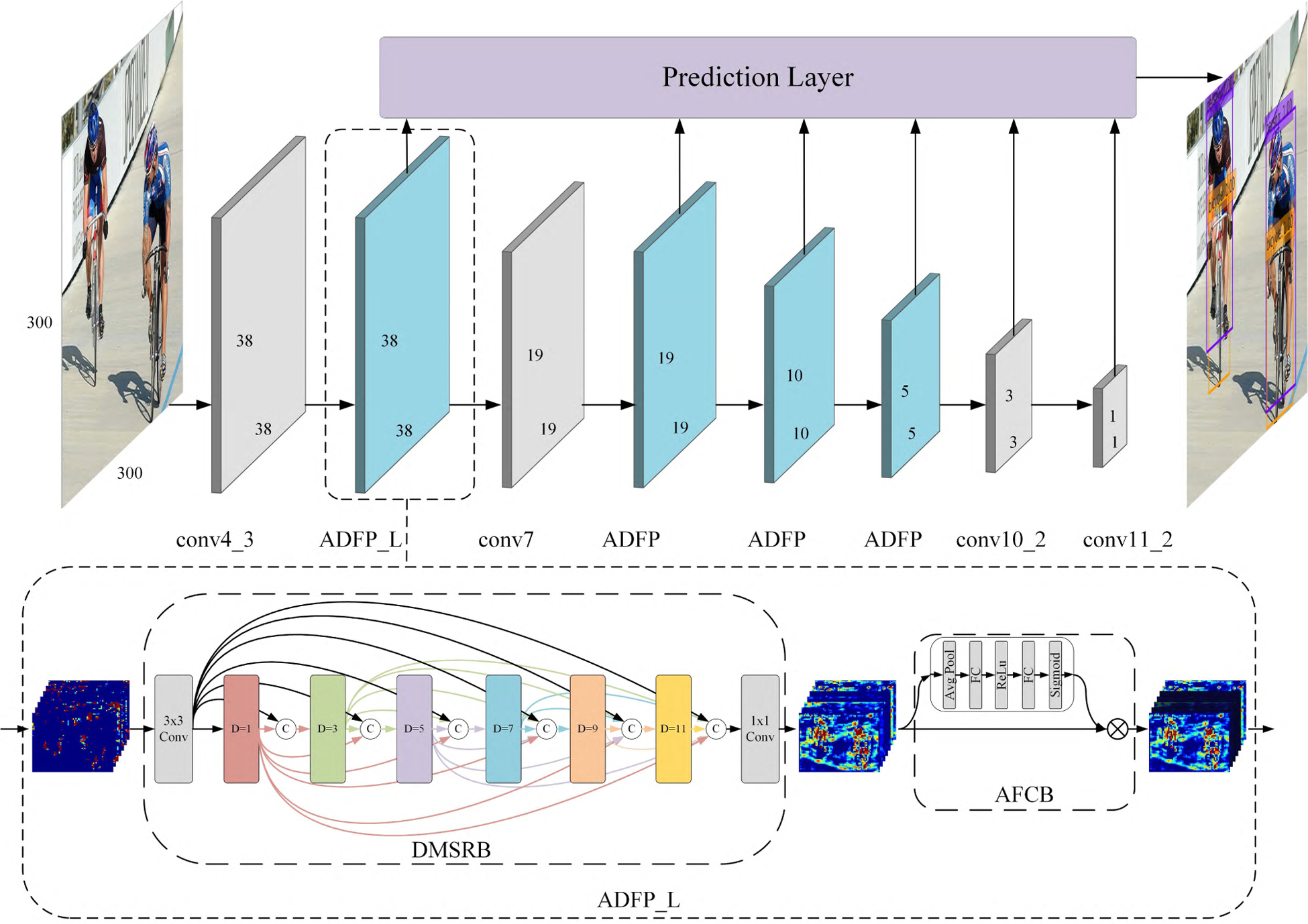
Architecture of the proposed adaptively dense feature pyramid network (ADFPNet). The proposed ADFP module first produces dense features across multi scales and receptive fields; then the dense features are re-calibrated according to their contribution to the detection task. The proposed module is seamless connected to the conv4_3 and conv7 layer. We use larger atrous rate for the ADFP module after conv4_3 as its larger feature resolution (denoted as ADFP_L).

We propose an adaptively dense feature pyramid network (ADFPNet), which is based on SSD framework with a novel adaptively dense feature pyramid (ADFP) module. The ADFP module is composed of two components including the dense multi scales and receptive fields block and the adaptively feature calibration block, as shown in Fig. 1. We describe the details in the follow sections.

### A. DENSE MULTI SCALES AND RECEPTIVE FIELDS BLOCK (DMSRB)

**FIGURE 2.**
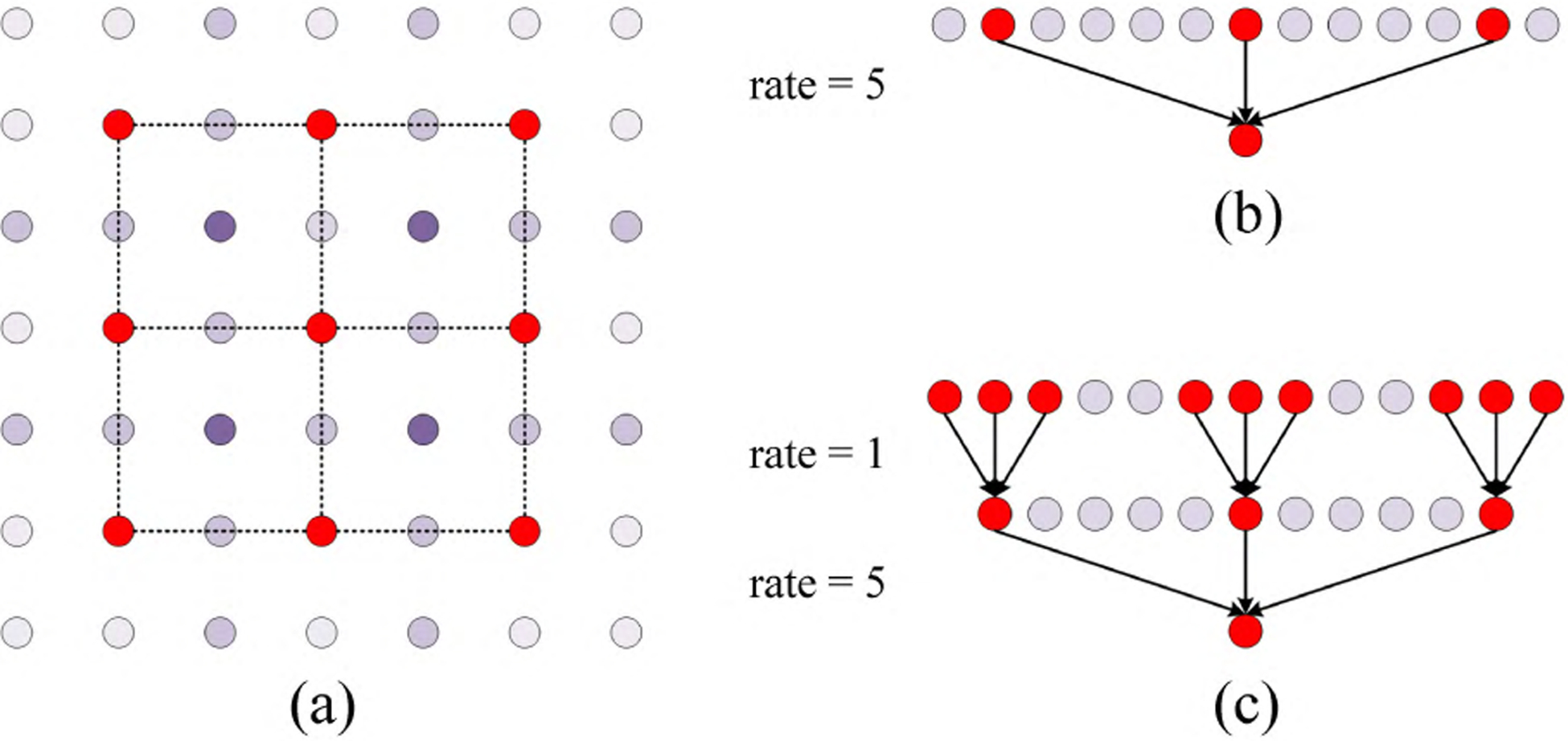
(a) A visual example of atrous convolution processing two-dimensional signals with atrous rate of 2. (b) An atrous convolution with an atrous rate of 5 sampling the signal of one dimension. (c) The atrous convolutions with atrous rates of 1 and 5 stacked in dense mode sampling the signal of one dimension.

Incorporating different scales and receptive fields has been proven to improve the detection accuracy [15], [17]. Thus, the purpose of this block is to generate dense features with multi-scales and different receptive fields. Inspired by the Densely connected Atrous Spatial Pyramid Pooling (DenseASPP), we design a dense multi scales and receptive fields block consisting of atrous convolution layers with different atrous rates to take full advantages of the multiple receptive field sizes and multiscale features. Compared to the DenseNet, we add atrous convolutions into the proposed module, which has shown to be effective for the feature extraction as it is widely used in the task of semantic segmentation. In two dimensional case, such as images, the atrous convolution operator responding each element *i* on the output *z* can be explained as follows:

(1) $$z[i]=\sum_{k=1}^{K}x[i+a\cdot k]w[k]$$

where *x* denotes the input feature maps, *a* represents the atrous rate, and *w*[*k*] corresponds the *k*-th parameter of the filter *w*. The atrous rate is the stride used for sampling the information from the input feature maps. Atrous convolution operation can be interpreted as inserting *a* − 1 zeros between two sequential filter elements along each spatial dimension to expand the filter by sampling the input *x*. One example of atrous convolution processing two-dimensional signals with atrous rate of 2 is visualized in Fig. 2(a). However, sampling the input feature *x* using large atrous rate would cause sparse information, as shown in Fig. 2(b), where the feature is extracted by an atrous convolution with an atrous rate of 5 in one dimension, while only 3 pixels contributing to the convolution calculation process leading to loss of information. Such problem can be alleviated by stacking larger atrous rate after smaller ones to gather information more intensively from more computational pixels. As shown in Fig. 2(c), through the stacking of the atrous convolutions with atrous rates of 1 and 5, 9 pixels in the one dimensional input participate in the convolution calculation, which is 3 times the number in the single atrous convolution calculation. When a two-dimensional signal is used as an input, a single 3 × 3 atrous convolution with an atrous rate of 5 aggregates 9 pixels while the stacked 3×3 atrous convolutions with atrous rates of 1 and 5 aggregate 81 pixels. On the other hand, the stacked atrous convolutions with different atrous rates produce image features with different scales, which helps for object detection. Specifically, to extract features in a dense mode, we stack the atrous convolutions in a dense connection mode, where each layer have access to ahead layers and all subsequent layers to obtain a dense receptive fields. The atrous rates are increasing as the layers in the module get deeper. Such computation of the dense atrous convolution layers can be formulated as:

(2) $$z_{d}=H_{d,a}\left(\left[z_{0},z_{1},\ldots ,z_{d-1}\right]\right)$$

where *zd* denotes the output of the *d*^*th*^ layer, [*z*_0_; *z*_1_, … , *zd*−1] corresponds to the concatenation of the outputs of the 0^*th*^, 1^*th*^, … , *d* − 1^*th*^ layers, and *H*_*a*_ denotes the *d*^*th*^ atrous convolution with atrous rate *a*. Obviously, the dense multi scales and receptive fields block can create a much denser feature pyramid for the reason that the atrous convolutions take all the previous layers’ outputs as input. Compared to the single atrous convolution, the atrous convolutions in dense mode with the same receptive fields can sample more information from the input. In other words, through the dense connection, more pixels are involved in the feature extraction process.

The details of our dense multi scales and receptive fields block are illustrated in Fig. 1. A feature transformation is used to aggregate and fuse the information in the input feature maps. The produced feature maps not only contain rich multi-scale semantic information about categories of objects, but also keep the fine details about the shape and location of objects.

### B. ADAPTIVELY FEATURE CALIBRATION BLOCK

The features extracted by the DMSRB contain extensive dense features from different scales and receptive fields. While given the assumption that not each channel in the dense feature contributes equally [40], thus some of the feature channels might be redundant. Such redundant features will obstacle the learning as well as the back-propogation. Thus, finding the useful features and depressing the redundant features become necessary. Thus, the adaptively feature calibration block (AFCB) is proposed to exploit the feature channel dependencies to calibrate the aggregated dense features by retaining on relatively important features and weakening relatively unrelated ones using the Squeeze-and-Excitation block [40]. Mathematically, let $x\in {\mathbb{R}}^{C\times H\times W}$ be a feature map that is passed from the DMSRB block and *F*_*c*_ be a transformation to produce a channel attention vector $c\in {\mathbb{R}}^{C\times 1\times 1}$ from input. The total adaptively feature calibration block can be expressed as follow:

(3) $$\bar{x}=F_{c}(x)\cdot x=c\cdot x$$

where $\bar{x}$ refers the calibrated features and is the element-wise multiplication. The channel vector *c* is created to clearly model the inter-channel dependency of the features. Each value in the *c* multiplies the corresponding feature channel by the broadcast mechanism along the channel dimension. In order to create *c* more efficiently, we first compress the input feature map *x* in the spatial dimension *H* × *W* by using average pooling, generating a channel descriptor: $p\in {\mathbb{R}}^{C\times 1\times 1}$. Then the channel descriptor *p* is sent into a two-layer perceptron neural network. To limit model complexity and increase computational efficiency, we first condense *p* to size $\frac{C}{r}\times 1\times 1$, then activate it in the hidden layer by ReLU activation function, and finally restore to size *C* × 1 × 1, then activate with Sigmoid function. The *r* is reduction rate used to flexibly adjust the channels of the descriptor *p*. The process of producing the channel attention vector *c* by this multi-layer perceptron (MLP) can be formulated as follow:

(4) $$c=\sigma \left(W_{1}\delta \left(W_{0}p\right)\right)$$

where *δ* denotes the ReLU activation function, the *σ* refers Sigmoid activation function, $W_{0}\in {\mathbb{R}}^{\frac{C}{r}\times C}$, and $W_{1}\in {\mathbb{R}}^{C\times \frac{C}{r}}$. The Sigmoid activation function projects the value2in range [0,1] with lower value depressing feature and higher value retaining feature.

### C. TRAINING LOSS

The overall objective loss function is a weighted sum of the classification loss (clc) and the localization loss (loc), as shown:

(5) $$L(x,c,l,g)=\frac{1}{N}\left(L_{clc}(x,c)+L_{loc}(x,l,g)\right)$$

where N is the number of matched default boxes. If *N* = 0, we set the loss to 0.

The classification loss is the softmax loss over multiple classes confidences (*c*).

(6) $$L_{clc}(x,c)=-\sum_{i\in Pos}^{N}x_{ij}^{p}log\left({\hat{c}}_{i}^{p}\right)-\sum_{i\in Neg}log\left({\hat{c}}_{i}^{0}\right)$$

where, ${\hat{c}}_{i}^{p}=\frac{exp\left(c_{i}^{p}\right)}{\sum_{p}exp\left(c_{i}^{p}\right)}$.

The localization loss is a Smooth L1 loss between the predicted box (*l*) and the ground truth box (*g*) parameters. Similar to Faster R-CNN, we regress to offsets for the center (*cx, cy*) of the default bounding box (*d*) and for its width (*w*) and height (*h*).

(7) $$L_{loc}(x,l,g)=\sum_{i\in Pos}^{N}\sum_{m\in \{cx,cy,w,h\}}x_{ij}^{k}S_{L1}\left(l_{i}^{m}-{\hat{g}}_{j}^{m}\right)$$

in which

(8) $${\hat{g}}_{j}^{cx}=\left(g_{j}^{cx}-d_{i}^{cx}\right)/d_{i}^{w}\;{\hat{g}}_{j}^{cy}=\left(g_{j}^{cy}-d_{i}^{cy}\right)/d_{i}^{h}\;{\hat{g}}_{j}^{w}=\text{log}\left(\frac{g_{j}^{w}}{d_{i}^{w}}\right)\;{\hat{g}}_{j}^{h}=\text{log}\left(\frac{g_{j}^{h}}{d_{i}^{h}}\right)$$

and

(9) $$S_{L1}=\left\{\begin{array}{ll} 0.5x^{2} & \left|x\right|\leq 1\\ \left|x\right|-0.5 & otherwise \end{array}\right.$$

### D. DETAILS OF NETWORK ARCHITECTURE

The motivation of the proposed adaptively dense feature pyramid network (ADFPNet) is to remedy the scale variation of object instances in object detection task with different receptive field sizes. To inherit the merits of SSD in accuracy and speed, we construct a feed-forward convolutional network that reuses the pyramidal feature hierarchy to produce category scores and box offsets for a fixed-size set of pre-set bounding boxes with ADFP module. Then the non-maximum suppression (nms) is followed to filter out most boxes to obtain the final detection results. The whole structure of ADFPNet is showed in Fig. 1.

#### 1) BACKBONE

To fairly compare with the original SSD, we choose the VGG-16 network, pre-trained on the ILSVRC dataset [41] for high quality image classification, as backbone. It is noticed that other backbone such as ResNet50 or ResNet 101 is also be the alternative candidates for the backbone. Due to differences in classification tasks and object detection tasks, we remove the final classification layers and add corresponding convolutional layers with sub-sampling parameters in VGG-16 to meet our needs.

#### 2) PYRAMIDAL FEATURE HIERARCHY

The original SSD uses the multi-scale feature maps with different resolutions from different layers, including conv4_3, conv7, conv8_2, conv9_2, conv 10_2, and conv11_2, to predict both locations and confidences of objects at vastly different scales, which is called pyramidal feature hierarchy. In our networks, we keep the pyramidal feature hierarchy but with different configurations using the proposed ADFP module as in Fig. 1. Firstly, we place the ADFP module after conv4_3 and conv7 layer. Features from these two layers are first processed then send to the prediction layer and successive layers. Secondly, we replace the conv8_x and conv9_x layers in the original SSD with an ADFP module respectively to produce more dense and semantic rich information. All the ADFP modules consist of a cascade of atrous convolution layers with atrous rates of 1, 2, 3, 4, and 5 except the one after conv4_3, where the atrous rates are 1, 3, 5, 7, 9, and 11. The reason is conv4_3 has larger feature map resolution and need larger atrous rate to capture the large receptive field. We indicate it as ADFP_L module, as shown in Fig. 1.

## IV. DATA AND EXPERIMENTS

We have conducted extensive experiments on three widely used benchmarks, namely Pascal VOC 2007, VOC 2012 [42], and MS COCO [43] datasets. The Pascal VOC datasets have 20 object categories, which are the subset of that in MS COCO including 80 object categories. VOC 2007 consists of 5,011 images as trainval set and 4,952 images as test set with all annotations available. In VOC 2012, the researchers annotate trianval set (11,540 images) and leave the test set (10,991 images) annotations unavailable. We split COCO dataset into train set (118k iamges), val set (5k images), and test set (41K iamges), which is much larger than the Pascal VOC datasets. The details on each dataset are described below.

### A. PASCAL VOC 2007

In this experiment, all the methods are trained on VOC 07 + 12 trainval set, the union of VOC 2007 trainval set and VOC 2012 trainval set, and tested on the VOC 2007 test set. In VOC 2007, the positive predicted bounding box, whose Intersection over Union (IoU) with the ground truth is higher than 0.5, is sent to predict the final results. We trian our method for 350 epochs using SGD with a “warm-up” strategy. Applying the “warm-up” strategy, we ramp up the learning rate from 10^−6^ to 4 × 10^−3^ at the first 5 epochs, and then multiply it by 0.1 at 200, 250, and 300 epochs. Referring to [13], we set the default batch size at 32, the weight decay to 5 × 10^−4^, and the momentum to 0.9 in the training. Due to the memory constraint, we halve the batch size and learning rate when training using 512 × 512 input, and keep the other settings unchanged.

### B. PASCAL VOC 2012

In this experiment, we train our ADFPNet on the union of VOC 2007 trainval set and test sets, and VOC 2012 trainval set (VOC 07 + +12), then submit the prediction results to the public evaluator. Considering the increase of training set, we adjust the total number of training epochs to 400. We set the learning rate to 4^−3^ after the same “warm-up” strategy followed VOC 2007, and divide it by 10 at 250, 300, and 350 epochs. The other training setting used in VOC 2007 are kept.

### C. MS COCO

To further validate our method, we conduct experiments on MS COCO dataset, which is a larger and more challenging dataset, and submit the prediction results of test-dev (20k images), which is a subset of the test set, to the official evaluation server to produce the mean Average Precision (mAP). MS COCO uses another evaluation metric different from VOC. The average mAP overing 10 different IoU thresholds from 0.5 to 0.95 is applied to evaluate the performance of the detection methods more comprehensively. APs with IOU thresholds of 0.5 and 0.75 are two other important evaluation indicators in COCO. In addition, COCO divides the object instances into large (area *>* 96^2^), medium (32^2^ *<* area *<* 96^2^), and small (area *<* 32^2^) according to the number of pixels in the segmentation mask to produce the corresponding APs. The training is conducted on the 2017 train set, which is exactly the same as the original public trainvel35k set as reported in the official website. We set the batch size to 32 in training and still apply the “warmup” strategy increasing the learning rate from 10^−6^ to 2 × 10^−3^ at the first 5 epochs. We continue to train the method with 2 × 10^−3^ learning rate for 95 epochs, then decay it to 2 × 10^−4^, 2 × 10^−5^, and 2 × 10^−6^ for another 50, 30, and 20 epochs, respectively. Referring to SSD, we reduce the size of the default anchor boxes while keeping the other settings same as in VOC since the size of object instances is smaller than that in VOC. Similarly, for the memory issue, we halve the batch size and learning rate for 512 × 512 input, increasing 20 epochs for the learning rate of 1 × 10^−3^.

## V. RESULT

### A. PASCAL VOC 2007

#### 1) QUANTITATIVE RESULT

**FIGURE 3.**
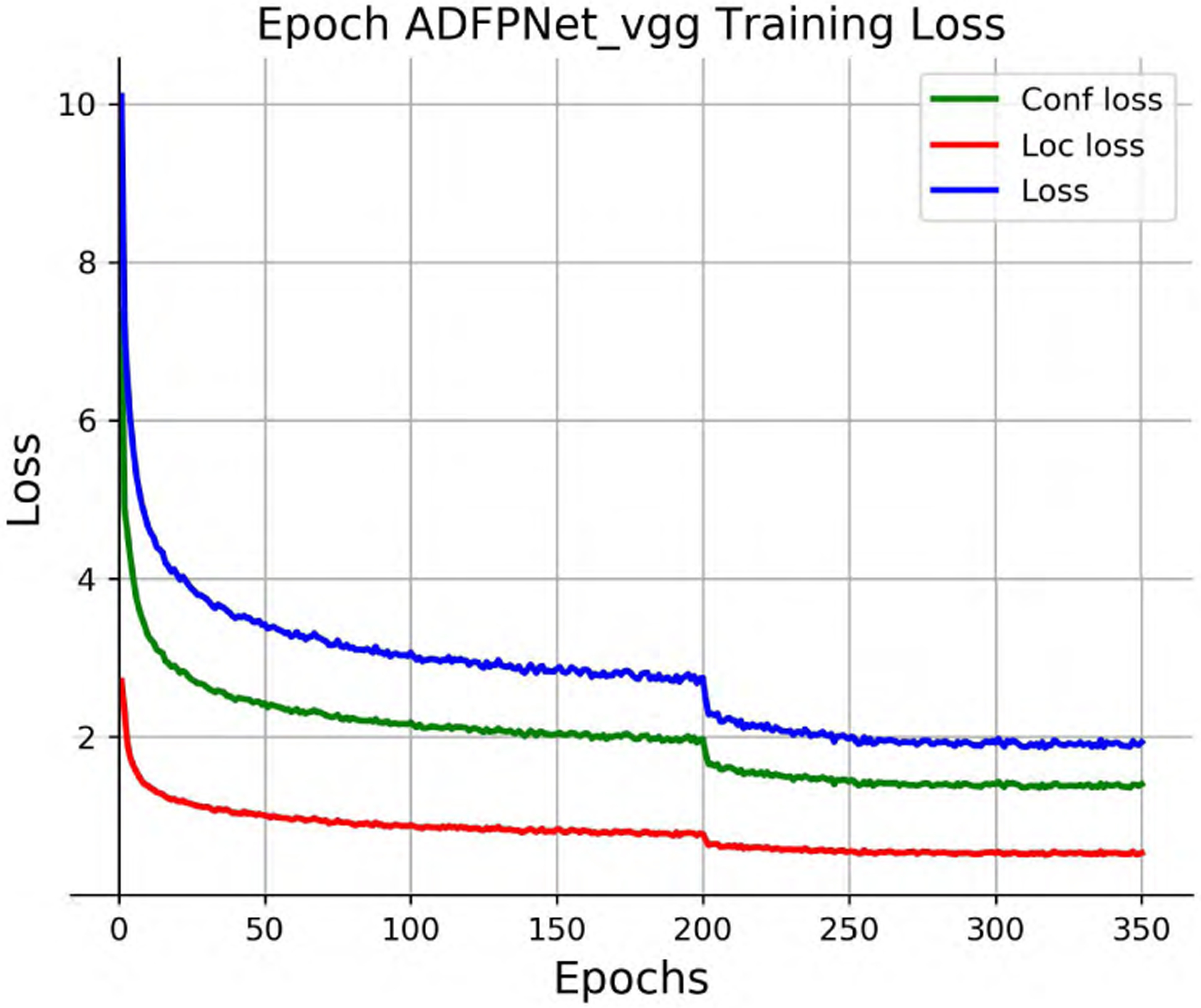
Training loss of ADFPNet300 on VOC 07 + 12 trainval set. Conf loss curve signifies the confidence loss. Loc loss curve signifies the localization loss. Loss denotes the total loss of confidence loss and localization loss. The horizontal axis represents the training epochs.

**FIGURE 4.**
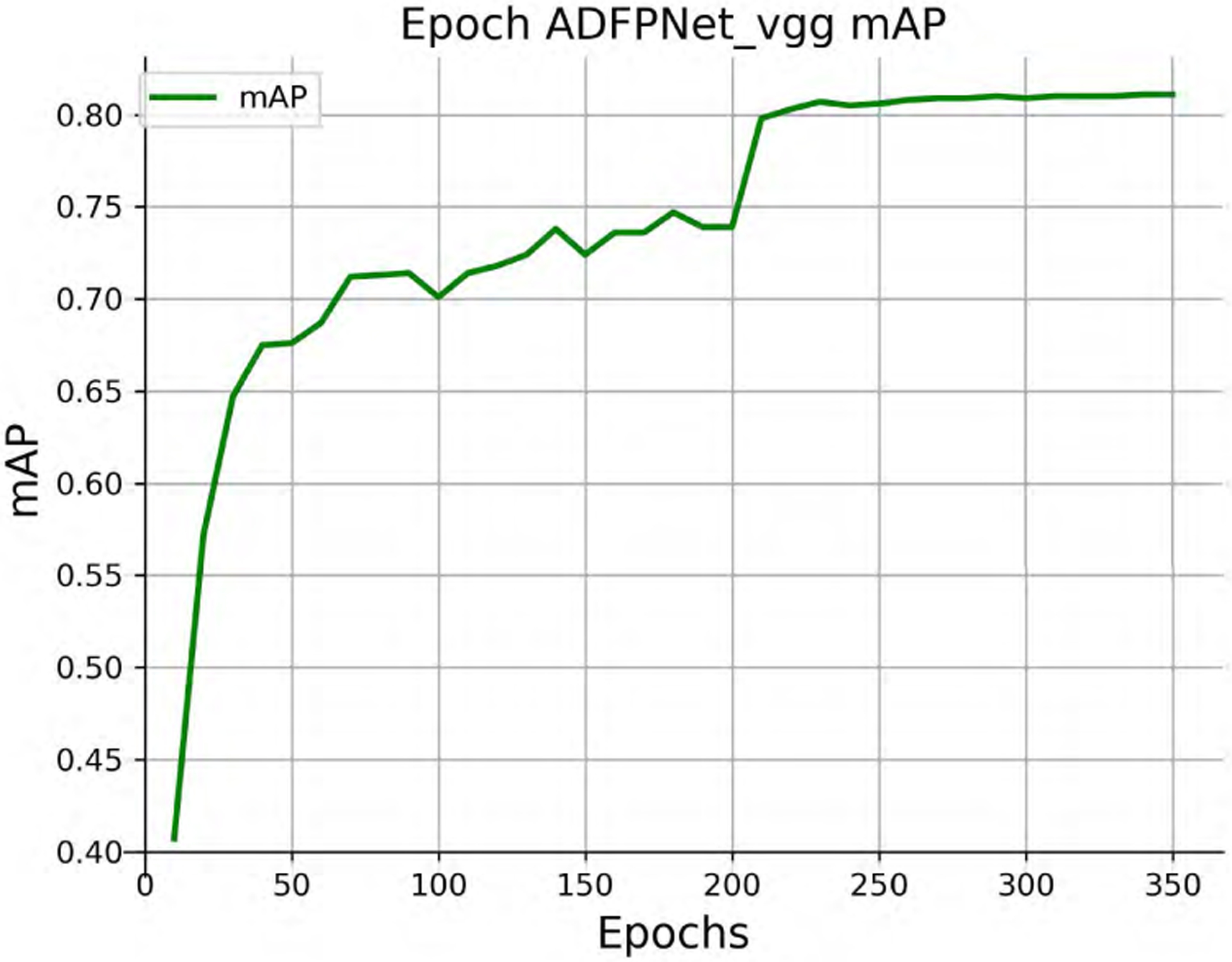
The mAP curve of ADFPNet300 trained on VOC 07 + 12 trainval set. It is tested on the VOC 2007 test set. The horizontal axis represents the training epochs.

**FIGURE 5.**
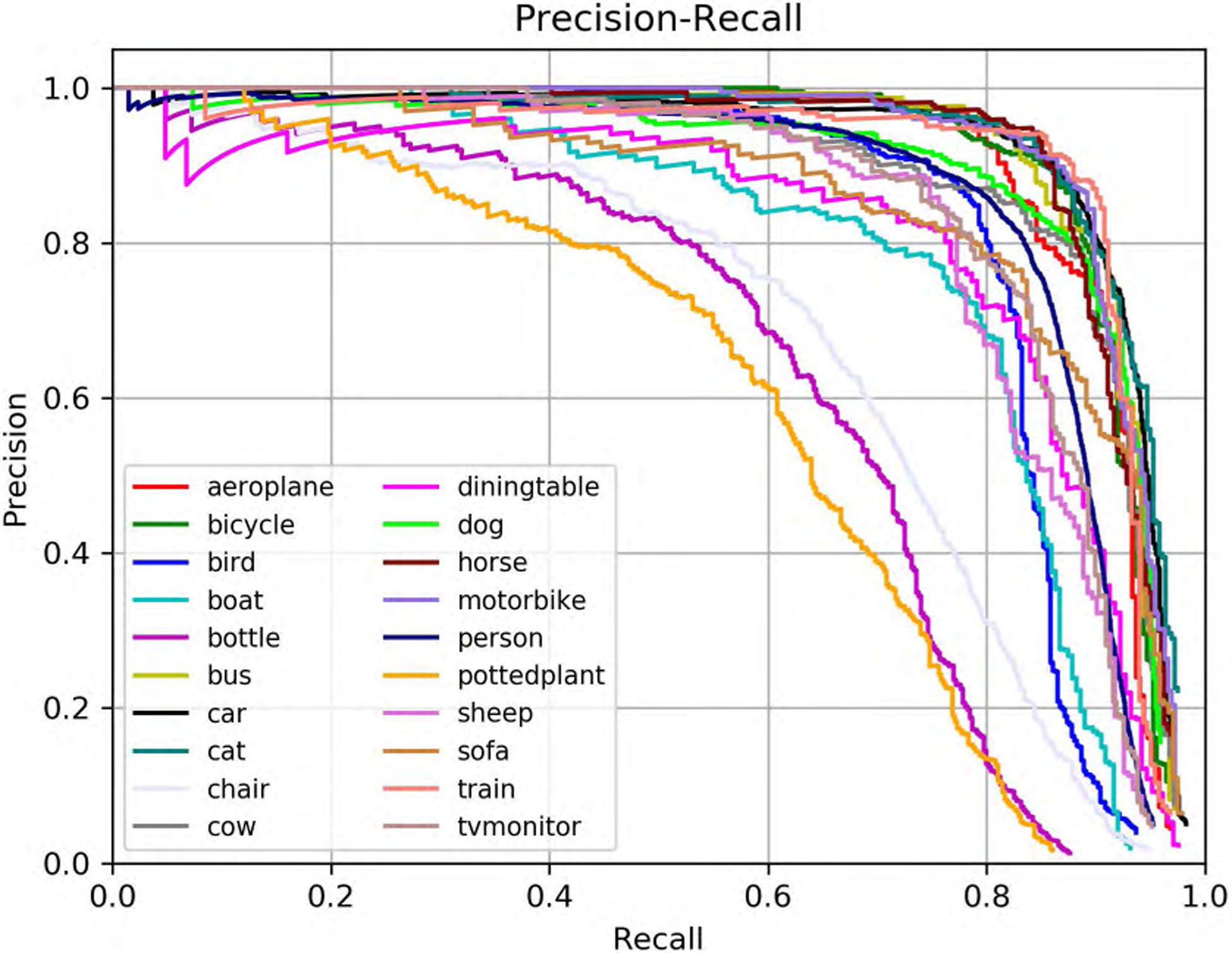
The precision-recall curve of ADFPNet300 tested on the VOC 2007 test set. It is trained on on VOC 07 + 12 trainval set.

Table 1 shows the performance comparison of ADFPNet with the state-of-the-art methods. The results of SSD300 and SSD512 are enhanced by using a “zoom in” operation to produce random crops as training examples. Our ADFPNet fed with low resolution input 300 × 300 achieves 81.1% mAP without any bells and whistles, which outperforms the SSD300 (77.2%) by a large margin and even exceeds the SSD512 (79.8%) in performance. It should be noticed that our ADFPNet is the first method obtaining above 81% mAP with such low resolution input as we known. By increasing the input size to 512 × 512, the performance of our method is further improved to 82.5% mAP, which exhibits the best mAP among the most advanced VGG-16 based methods (e.g., RefineDet, RFBNet, and PFPNet-R, etc). Our ADFPNet512 surpasses most of the two-stage object detectors including ResNet-101 based Faster RCNN and R-FCN, and shows the result similar to CoupleNet [48], which designs different coupling strategies and normalization ways to couple the global structure with local parts for object detection. Note that, two-stage object detectors typically use high-resolution images (i.e., ~ 600 × 1000) as input and use ResNet-101 as the base network, which yield higher detection performance but greatly increase the inference time as we all know. Compared to the real-time methods such as SSD, YOLOv2, RefineDet, and RFBNet, ADFPNet not only exceeds them in performance, but also is on par with them in inference speed. In order to make our training process more intuitive, the loss and mAP curves of ADFPNet300 during training are shown in Fig. 3 and Fig. 4, respectively. The mAP is evaluated on the VOC 2007 test set every 10 epochs. Moreover, the precision-recall curve of ADFPNet300 tested on the VOC 2007 test set is shown in Fig. 5.

#### 2) QUALITATIVE RESULT

**FIGURE 6.**
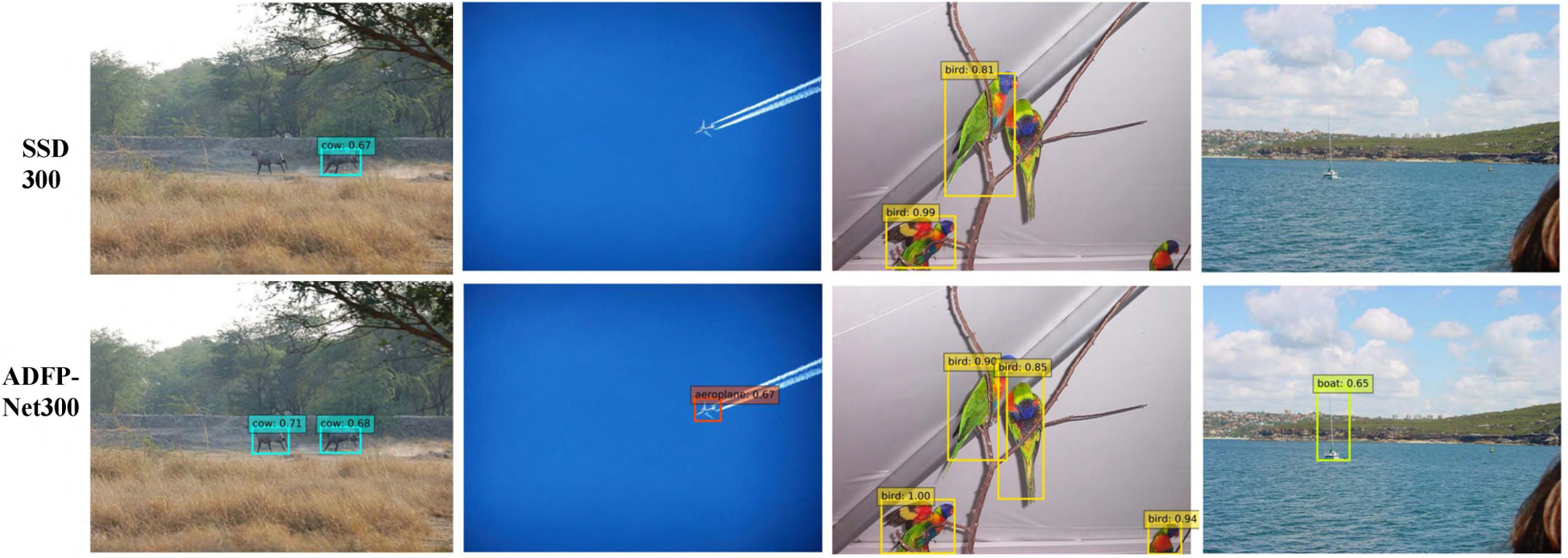
The detection results of proposed ADFPNet300 compared with SSD300 [13]. Rectangle areas represent the detected object with confidence score.

The detection results across multi-objects and different scales on VOC 2007 test set compared with the SSD300 [13] are shown in Fig. 6. It suggested that the SSD method missed objects in very small scale while the proposed method can capture and detect them successfully, which contributes to the the proposed module.

#### 3) FEATURE MAP BEFORE AND AFTER CALIBRATION

**FIGURE 7.**
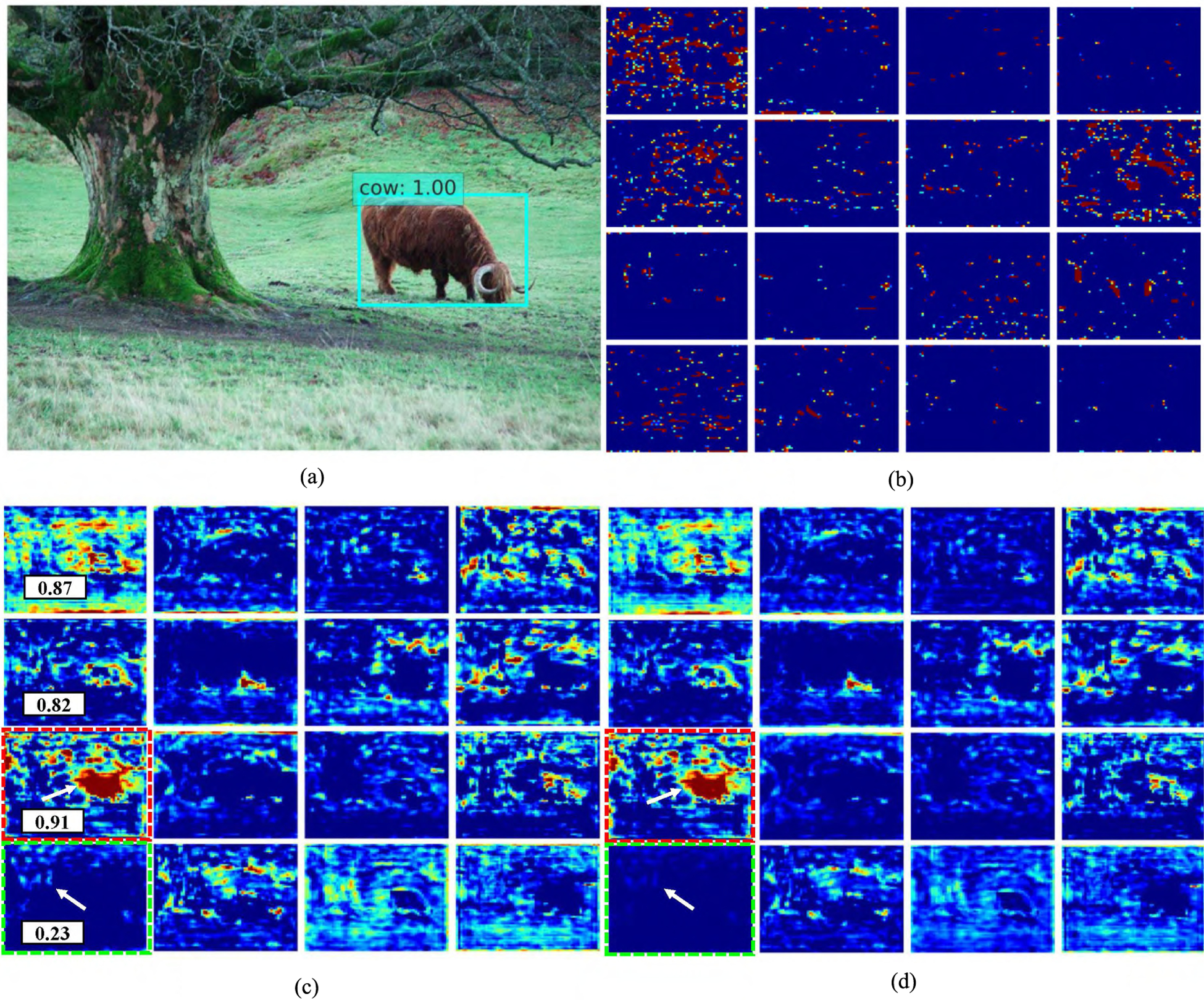
The feature maps of ADFPNet512 before and after self-feature calibration. (a) shows a detection results; (b) shows the feature map from Conv4_3 layer of channel 192 to 207 generated by SSD; (c) shows the feature map generated by our method before feature calibration and (d) shows the corresponding feature of (c) after calibration. The numbers in (c) show the relative feature weights calculated by the adaptively feature calibration block. The red-dot rectangle represents weighted more while the green-dot rectangle is the feature which is depressed. Best viewed in color.

**FIGURE 8.**
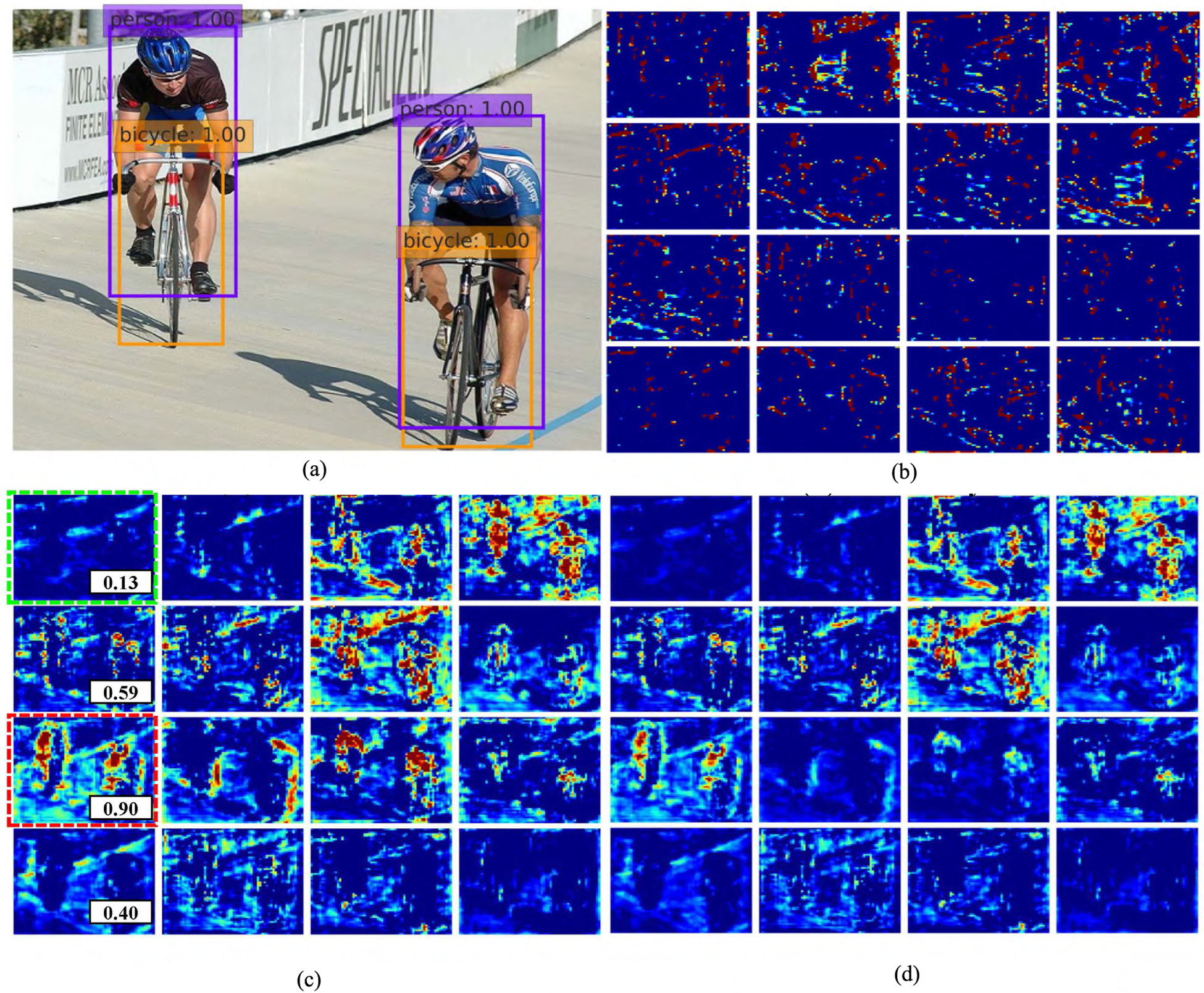
The feature maps of ADFPNet512 before and after self-feature calibration. (a) shows a detection results; (b) shows the feature map from Conv4_3 layer of channel 288 to 303 generated by SSD; (c) shows the feature map generated by our method before feature calibration and (d) shows the corresponding feature of (c) after calibration. The numbers in (c) show the relative feature weights calculated by the adaptively feature calibration block. The red-dot rectangle represents weighted more while the green-dot rectangle is the feature which is depressed. Best viewed in color.

We also show the qualitative results of ADFPNet512 on the feature map before and after self-calibration in Fig. 7 and 8. It suggested that the feature calibration block did depress the features which offers less or sparse information by learning a lower weight (shown in green dot rectangle in Fig. 7 and 8(c)) and assigning a higher weight for the features containing useful information (shown in red dot rectangle in Fig. 7 and 8(c)).

### B. PASCAL VOC 2012

Table 2 shows the detection accuracy of the proposed ADFPNet with the other state-of-the-art frameworks. To better demonstrate the effectiveness of our ADFPNet, we separately report the results of each category in VOC 2012 test set. Compared with the frameworks using the similar input size, ADFPNet300 produces the best mAP of 79.0%, which has even surpassed most two-stage frameworks using much deeper base network (i.e., ResNet-101 [3]) and larger input size around 1000 × 600. When the input size to 512 × 512, ADFPNet512 achieves best mAP of 81.9%, outperforming the most recently proposed frameworks aiming to detect the multi-scale objects by a large margin (e.g., 80.3% mAP of DES512 [35] and 80.0% mAP of DFPR512 [34]). To the best of our knowledge, ADFPNet is the first framework to obtain performance above 81% mAP on VOC 2012 without any bells and whistles.

### C. MS COCO

Table 3 shows the comparison of our method and the other state-of-the-art methods. ADFPNet300 produces 31.8% mAP, which outperforms the other VGG-16 based detectors with the same input size of 300 × 300. It is also noticed that the accuracy of proposed ADFPNet300 is higher than RefineDet320 by 2.4%, which designs the anchor refinement module (ARM) to filter out the negative anchors and coarsely adjust the positive anchors with slightly larger input images. The accuracy of ADFPNet300 even exceeds R-FCN based on ResNet-101 backbone and is similar to RetinaNet400 which uses ResNet-101 as backbone and a 400 × 400 input size. It should be noticed that our method is much better than the recent advanced one-stage detectors which try to include multi-scale context information such as DFPR [34], RFBNet [15], and PFPNet-R [17]. Furthermore, when testing under input image size of 512 × 512, the performance of ADFPNet512 can further improve to 36.4%, which outperforms most of one-stage methods except ResNet-101-FPN based RetinaNet800*, which adopted scale jitter, used the 800 × 800 input image, and was trained for 1.5× longer than RetinaNet500. Compared with the two-stage methods, ADFPNet512 surpasses most of them except Faster R-CNN w/ TDM and Deformable R-FCN with complex backbone and large input size (i.e.,1000 × 600).

**FIGURE 9.**
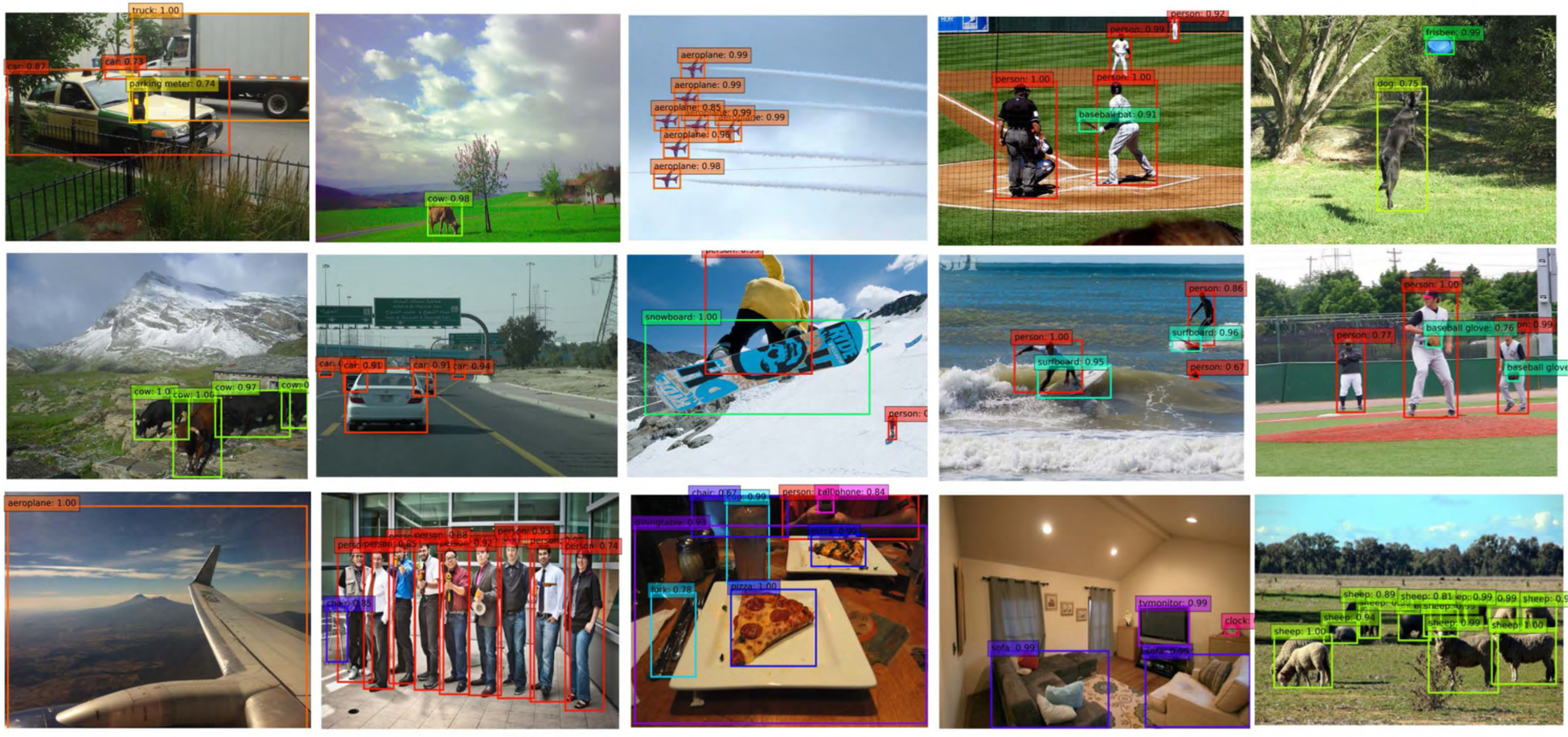
The detection results of ADFPNet512 on the MS COCO test-dev set. The training data is COCO 2017 train set.

Our proposed ADFPNet also shows excellent performance on small object detection in COCO dataset. In COCO, approximately 41% of objects are small while only 24% are large as small object detection is still a fundamental problem in computer vision. As shown in Table 4, ADFPNet300 and ADFPNet512 achieve 12.6% and 19.2% mAPs, respectively on the small objects which demonstrates the effeciency and advantage of proposed method. Moreover, ADFPNet512 achieves the best AP on small objects among the VGG-based detectors and even better than most of ResNet backbone based detectors. We show the detection results of ADFPNet512 on the MS COCO test-dev set in the Fig. 9.

## VI. DISCUSSION

### A. ARCHITECTURE ABLATION AND STUDY

We conduct experiments on the union of VOC 2007 and VOC 2012 trainval sets to exploit the influence of ADFP module, VOLUME 7, 2019 ADFP_L module, adaptively feature calibration block, and more default boxes. The accuracy is evaluated on VOC 2007 test set, as shown in Table 4. In all the experiment, the input image size is set to 300 × 300 and all the other hyperparameters are set to be the same.

#### 1) ADAPTIVELY FEATURE CALIBRATION BLOCK (ROW 2)

To verify the effectiveness of adaptively feature calibration block, we construct a variant network by removing it. As listed in Table 4, the variant increases the performance by 3.5% mAP as compared to the baseline. With the adaptively feature calibration block, the mAP is further improved from 80.7% to 81.1%.

#### 2) MORE DEFAULT BOXES (ROW 3)

In the original SSD, the feature map of conv4_3 contains fine details, which is critical for location, but lacks strong semantic information, which is used for classification. Therefore, only 4 default anchor boxes are associated at each location of conv4_3, conv10_2, and conv11_2, while 6 default anchor boxes are associated at each location of the other layers. We sent the feature maps from conv4_3 into our ADFP_L module to produce a feature map containing rich details and semantic information, wihch is necessary for detecting small object instances. Thus, in order to improve the performance, especially for small instances, we set 6 default boxes, adding aspect ratios of $\frac{1}{3}$ and 3, on the feature map from ADFP_L module, which has no effect on the original SSD as mentioned in [15]. As shown in the third and fifth rows in Table 4, adding more default boxes increases the mAP from 80.3% to 81.1%.

#### 3) ADFP MODULE

To demonstrate the effectiveness of ADFP module, we redesign a simple network only with the ADFP module and use the original SSD with new data augmentation as a baseline. The SSD obtains the detection performance of 77.2% as shown in the first row of Table 4. Obviously, with the simple introduction of our ADFP module, this performance is improved to 80.2%. The 3% gain fully demonstrates that our module, which extracts the features with different receptive fields in a dense way, can significantly boost detection performance.

Due to the feature map produced from conv4_3 is much bigger than the others, we correspondingly adjust the atrous rates to constitute a new module, defined as ADFP_L. As can be seen in the fourth and fifth rows of Table 4, the adding of ADFP_L module network further increases the performance by 0.9% mAP as compared to the network only with ADFP module. This probably contributes to the sufficient contextual field from conv4_3 when using larger atrous rates.

### B. INFERENCE TIME STUDY

To quantitatively evaluate inference time, we test SSD and ADFPNet with batch size 1 on our machine with an NVIDIA 1080ti, CUDA 9.0 and cuDNN v7 to compare fairly. All the methods are trained on the VOC 07 + 12 trianval set and evaluated on the VOC 2007 test set with 300 × 300 input size. We report all the results in Table 4. The ADFPNet300 without ADFP_L module outperforms the original SSD300 with a large margin (80.2% vs 77.2%), although it spends a little extra time (15 ms/img vs 8 ms/img). The addition of the ADFP_L module consumes almost no extra time but improves performance by 0.9%. Finally, our framework has a 3.9% accuracy gain compared to SSD with an FPS of 62.5. It strongly proves that our proposed ADFP_L and ADFP module significantly help promote the detection performance while meeting the needs of real-time detection (30 frames per second or better), as mentioned in [57] and [29].

## VII. CONCLUSION

We present a novel adaptively dense feature pyramid network (ADFPNet) for object detection under the Single Shot MultiBox Detector (SSD) framework. The proposed network is able to detect objects across different scales by extracting feature maps with dense multi scales and receptive fields. Extensive experiments have been conducted on several public benchmarks, Pascal VOC 2007, Pascal VOC 2012, and MS COCO to demonstrate the efficiency of our method, which achieves the state-of-the-art performance without any bells and whistles. Moreover, the proposed method also achieves a good balance between detection accuracy and inference speed.

## Figures and Tables

**TABLE 1.** Detection results on PASCAL VOC 2007 test set.

Method	Data	Backbone	Input Size	Boxes	FPS	mAP
Fast RCNN [24]	07+12	VGG-16	~ 600 × 1000	~2000	0.5	70.0
Faster RCNN [25]	07+12	VGG-16	~ 600 × 1000	300	7	73.2
Faster RCNN [25]	07+12	ResNet-101	~ 600 × 1000	300	2.4	76.4
OHEM [44]	07+12	VGG-16	~ 600 × 1000	300	7	74.6
HyperNet [45]	07+12	VGG-16	~ 600 × 1000	100	0.88	76.3
ION [46]	07+12	VGG-16	~ 600 × 1000	4000	1.25	76.5
MR-CNN [47]	07+12	VGG-16	~ 600 × 1000	250	0.03	78.2
R-FCN [26]	07+12	ResNet-101	~ 600 × 1000	300	9	80.5
CoupleNet [48]	07+12	ResNet-101	~ 600 × 1000	300	8.2	**82.7**
RON384 [49]	07+12	VGG-16	384 × 384	30600	15	75.4
YOLO [29]	07+12	GoogleNet	448 × 448	98	45	63.4
YOLOv2 [30]	07+12	Darknet-19	544 × 544	845	40	78.6
SSD300 [13]	07+12	VGG-16	300 × 300	8732	46	77.2
SSD512 [13]	07+12	VGG-16	512 × 512	24564	19	79.8
DSOD300 [50]	07+12	DS/64-192-48-1	300 × 300	8732	17.4	77.7
DSSD321 [32]	07+12	ResNet-101	321 × 321	17080	9.5	78.6
DSSD513 [32]	07+12	ResNet-101	513 × 513	43688	5.5	81.5
RefineDet320 [33]	07+12	VGG-16	320 × 320	6375	40.3	80.0
RefineDet512 [33]	07+12	VGG-16	512 × 512	16320	24.1	81.8
DES300 [35]	07+12	VGG-16	300 × 300	-	76.8	79.7
DES512 [35]	07+12	VGG-16	512 × 512	-	31.7	81.7
DFPR300 [34]	07+12	VGG-16	300 × 300	-	39.5	79.6
DFPR512 [34]	07+12	VGG-16	512 × 512	-	-	81.1
RFBNet300 [34]	07+12	VGG-16	300 × 300	11620	83	80.5
RFBNet512 [34]	07+12	VGG-16	512 × 512	32756	38	82.2
PFPNet-R320 [17]	07+12	VGG-16	300 × 300	6375	33	80.7
PFPNet-R512 [17]	07+12	VGG-16	512 × 512	16320	24	82.3
ADFPNet300	07+12	VGG-16	300 × 300	11620	62.5	81.1
ADFPNet512	07+12	VGG-16	512 × 512	32756	43.5	**82.5**

**07+12** signifies the combined set of 07 trainval and 12 trainval. It should be noted that the results of SSD300 and SSD512 are improved by the new expansion data augmentation method.

**TABLE 2.** Detection results on PASCAL VOC 2012 test set.

Method	Backbone	mAP	aero	bike	bird	boat	bottle	bus	car	cat	chair	cow	table	dog	horse	mbike	person	plant	sheep	sofa	train	tv
Faster RCNN [25]	ResNet-101	73.8	86.5	81.6	77.2	58.0	51.0	78.6	76.6	93.2	48.6	80.4	59.0	92.1	85.3	84.8	80.7	48.1	77.3	66.5	84.7	65.6
ION [46]	VGG-16	76.4	87.5	84.7	76.8	63.8	58.3	82.6	79.0	90.9	57.8	82.0	64.7	88.9	86.5	84.7	82.3	51.4	78.2	69.2	85.2	73.5
R-FCN [26]	ResNet-101	77.6	86.9	83.4	81.5	63.8	62.4	81.6	81.1	93.1	58.0	83.8	60.8	92.7	86.0	84.6	84.4	59.0	80.8	68.6	86.1	72.9
RON384++ [49]	VGG-16	75.4	86.5	82.9	76.6	60.9	55.8	81.7	80.2	91.1	57.3	81.1	60.4	87.2	84.8	84.9	81.7	51.9	79.1	68.6	84.1	70.3
YOLO [29]	GoogleNet	57.9	77.0	67.2	57.7	38.3	22.7	68.3	55.9	81.4	36.2	60.8	48.5	77.2	72.3	71.3	63.5	28.9	52.2	54.8	73.9	50.8
YOLOv2 [30]	Darknet-19	73.4	86.3	82.0	74.8	59.2	51.8	79.8	76.5	90.6	52.1	78.2	58.5	89.3	82.5	83.4	81.3	49.1	77.2	62.4	83.8	68.7
SSD300 [13]	VGG-16	75.8	88.1	82.9	74.4	61.9	47.6	82.7	78.8	91.5	58.1	80.0	64.1	89.4	85.7	85.5	82.6	50.2	79.8	73.6	86.6	72.1
SSD512 [13]	VGG-16	78.5	90.0	85.3	77.7	64.3	58.5	85.1	84.3	92.6	61.3	83.4	65.1	89.9	88.5	88.2	85.5	54.4	82.4	70.7	87.1	75.6
DSSD321 [32]	ResNet-101	76.3	87.3	83.3	75.4	64.6	46.8	82.7	76.5	92.9	59.5	78.3	64.3	91.5	86.6	86.6	82.1	53.3	79.6	75.7	85.2	73.9
DSSD513 [32]	ResNet-101	80.0	**92.1**	86.6	80.3	68.7	58.2	84.3	85.0	**94.6**	63.3	85.9	65.6	**93.0**	88.5	87.8	86.4	57.4	85.2	73.4	87.8	76.8
DSOD300 [50]	DS/64-192-48-1	76.3	89.4	85.3	72.9	62.7	49.5	83.6	80.6	92.1	60.8	77.9	65.6	88.9	85.5	86.8	84.6	51.1	77.7	72.3	86.0	72.2
R-SSD300 [51]	VGG-16	76.4	88.0	83.8	74.8	60.8	48.9	83.9	78.5	91.0	59.5	81.4	66.1	89.0	86.3	86.0	83.0	51.3	80.9	73.7	86.9	73.8
RUN300 [52]	VGG-16	77.1	88.2	84.4	76.2	63.8	53.1	82.9	79.5	90.9	60.7	82.5	64.1	89.6	86.5	86.6	83.3	51.5	83.0	74.0	87.6	74.4
RUN512 [52]	VGG-16	79.8	90.0	87.3	80.2	67.4	62.4	84.9	85.6	92.9	61.8	84.9	66.2	90.9	89.1	88.0	86.5	55.4	85.0	72.6	87.7	76.8
StairNet [53]	VGG-16	76.4	87.7	83.1	74.6	64.2	51.3	83.6	78.0	92.0	58.9	81.8	66.2	89.6	86.0	84.9	82.6	50.9	80.5	71.8	86.2	73.5
RefineDet320 [33]	VGG-16	78.1	90.4	84.1	79.8	66.8	56.1	83.1	82.7	90.7	61.7	82.4	63.8	89.4	86.9	85.9	85.7	53.3	84.3	73.1	87.4	73.9
RefineDet512 [33]	VGG-16	80.1	90.2	86.8	81.8	68.0	**65.6**	84.9	85.0	92.2	62.0	84.4	64.9	90.6	88.3	87.2	87.8	58.0	86.3	72.5	88.7	76.6
DES300 [35]	VGG-16	77.1	88.5	84.4	76.0	65.0	50.1	83.1	79.7	92.1	61.3	81.4	65.8	89.6	85.9	86.2	83.2	51.2	81.4	**76.0**	88.4	73.3
DES512 [35]	VGG-16	80.3	91.1	87.7	81.3	66.5	58.9	84.8	85.8	92.3	64.7	84.3	67.8	91.6	**89.6**	88.7	86.4	57.7	85.5	74.4	89.2	77.6
DFPR300 [34]	VGG-16	77.5	89.5	85.0	77.7	64.3	54.6	81.6	80.0	91.6	60.0	82.5	64.7	89.9	85.4	86.1	84.1	53.2	81.0	74.2	87.9	75.9
DFPR512 [34]	VGG-16	80.0	89.6	87.4	80.9	68.3	61.0	83.5	83.9	92.4	63.8	85.9	63.9	89.9	89.2	88.9	86.2	56.3	84.4	75.5	**89.7**	**78.5**
ADFPNet300	VGG-16	79.0	90.4	86.8	78.1	67.4	54.4	85.1	82.1	92.4	65.0	84.6	67.5	90.0	88.7	86.8	85.4	55.7	84.4	73.2	87.8	74.7
ADFPNet512	VGG-16	**81.9**	**91.4**	**88.6**	**82.5**	**68.8**	**64.6**	**86.6**	**87.4**	**92.5**	**68.1**	**87.7**	**71.6**	**90.5**	**89.0**	**89.7**	**88.7**	**63.0**	**87.2**	**74.4**	**88.9**	**77.2**

Note that all frameworks in this table are trained on the PASCAL VOC dataset combined of 07 trainval + 07 test + 12 trainval. Result links are ADFPNet300: http://host.robots.ox.ac.uk:8080/anonymous/LCTQBI.html; ADFPNet512: http://host.robots.ox.ac.uk:8080/anonymous/4QPR7R.html.

**TABLE 3.** Detection results on COCO test-dev set.

Method	Data	Backbone	Avg.Precision, Iou:			Avg.Precision, Area:			Avg.Recall, #Dets:			Avg.Recall, Area:		
			0.5:0.95	0.5	0.75	S	M	L	1	10	100	S	M	L
Faster RCNN [25]	trainval	VGG-16	21.9	42.7	-	-	-	-	-	-	-	-	-	-
ION [46]	train	ResNet-101	23.6	43.2	23.6	6.4	24.1	38.3	23.2	32.7	33.5	10.1	37.7	53.6
OHEM++ [44]	trainval	VGG-16	25.5	45.9	26.1	7.4	27.7	40.3	-	-	-	-	-	-
R-FCN [26]	trainval	ResNet-101	29.9	51.9	-	10.8	32.8	45.0	-	-	-	-	-	-
CoupleNet [48]	trainval	ResNet-101	34.4	54.8	37.2	13.4	38.1	50.8	-	-	-	-	-	-
Faster RCNN+++ [3]	trainval	ResNet-101-C4	34.9	55.7	37.4	15.6	38.7	50.9	-	-	-	-	-	-
Faster R-CNN w/ FPN [14]	trainval35k	ResNet-101-FPN	36.2	**59.1**	39.0	18.2	39.0	48.2	-	-	-	-	-	-
Faster R-CNN w/ TDM [54]	trainval	Inception-ResNet	37.3	57.8	39.8	17.1	40.3	52.1	-	-	-	-	-	-
Deformable R-FCN [55]	trainval	Aligned-Inception-ResNet	37.5	58.0	40.8	19.4	40.1	**52.5**	-	-	-	-	-	-
YOLOv2 [30]	trainval35k	Darknet-19	21.6	44.0	19.2	5.0	22.4	35.5	20.7	31.6	33.3	9.8	36.5	54.4
SSD300 [13]	trainval35k	VGG-16	25.1	43.1	25.8	6.6	25.9	41.4	23.7	23.7	37.2	11.2	40.4	58.4
SSD512 [13]	trainval35k	VGG-16	28.8	48.5	30.3	10.9	31.8	43.5	26.1	39.5	42.0	16.5	46.6	60.8
RON384++ [49]	trainval	VGG-16	27.4	49.5	27.1	-	-	-	-	-	-	-	-	-
SSD321 [32]	trainval35k	ResNet-101	28.0	45.4	29.3	6.2	28.3	49.3	25.9	37.8	39.9	11.5	43.3	64.9
SSD513 [32]	trainval35k	ResNet-101	31.2	50.4	33.3	10.2	34.5	49.8	28.3	42.1	44.4	17.6	49.2	65.8
DSSD321 [32]	trainval35k	ResNet-101	28.0	46.1	29.2	7.4	28.1	47.6	25.5	37.1	39.4	12.7	42.0	62.6
DSSD513 [32]	trainval35k	ResNet-101	33.2	33.2	35.2	13.0	35.4	51.1	28.9	43.5	46.2	21.8	49.1	**66.4**
DSOD300 [50]	trainval	DS/64-192-48-1	29.3	47.3	30.6	9.4	31.5	47.0	27.3	40.7	43.0	16.7	47.1	65.0
DES300 [35]	trainval35k	VGG-16	28.3	47.3	29.4	8.5	29.9	45.2	25.6	38.3	40.7	14.1	44.7	62.0
DES512 [35]	trainval35k	VGG-16	32.8	53.2	34.6	13.9	36.0	47.6	28.4	43.5	46.2	21.6	50.7	64.6
RefineDet320 [33]	trainval35k	VGG-16	29.4	49.2	31.3	10.0	32.0	44.4	26.2	42.2	45.8	18.7	52.1	66.0
RefineDet512 [33]	trainval35k	VGG-16	33.0	54.5	35.5	16.3	36.3	44.3	28.3	46.4	**50.6**	**29.3**	**55.5**	66.0
DFPR300 [34]	trainval35k	VGG-16	28.4	48.2	29.1	-	-	-	-	-	-	-	-	-
DFPR512 [34]	trainval35k	VGG-16	31.5	50.9	32.2	-	-	-	-	-	-	-	-	-
RFBNet300 [15]	trainval35k	VGG-16	30.3	49.3	31.8	11.8	31.9	45.9	-	-	-	-	-	-
RFBNet512 [15]	trainval35k	VGG-16	33.8	54.2	35.9	16.2	37.1	47.4	-	-	-	-	-	-
PFPNet-R320 [17]	trainval35k	VGG-16	31.8	52.9	33.6	12.0	35.5	46.1	-	-	-	-	-	-
PFPNet-R512 [17]	trainval35k	VGG-16	35.2	57.6	37.9	18.7	38.6	45.9	-	-	-	-	-	-
RetinaNet400 [56]	trainval35k	ResNet-101	31.9	49.5	34.1	11.6	35.8	48.5	-	-	-	-	-	-
RetinaNet500 [56]	trainval35k	ResNet-101	34.4	53.1	36.8	14.7	38.5	49.1	-	-	-	-	-	-
RetinaNet800* [56]	trainval35k	ResNet-101-FPN	**39.1**	**59.1**	**42.3**	**21.8**	**42.7**	50.2	-	-	-	-	-	-
ADFPNet300	train2017	VGG-16	31.8	51.3	33.7	12.6	35.2	46.8	28.0	42.1	44.2	18.8	49.5	63.5
ADFPNet512	train2017	VGG-16	**36.4**	**56.9**	**38.8**	**19.2**	**41.1**	**49.3**	**30.7**	**47.5**	**50.2**	**28.7**	**54.9**	**66.4**

Note that **train2017** signifies the COCO 2017 train set which consists of the same exact images as trainval35k. RetinaNet800* adopted scale jitter and was trained for 1.5× longer than RetinaNet500 using input image size of 800 × 800.

**TABLE 4.** PASCAL VOC 2007 test detection results for ablation study.

Method	Backbone	ADFP	ADFP_L	AFCB	More anchors	Time(ms/img)	mAP
SSD300	VGG-16	-	-	-	-	8.0	77.2
ADFPNet300	VGG-16	✓	✓	-	✓	15.0	80.7
ADFPNet300	VGG-16	✓	✓	✓	-	16.0	80.3
ADFPNet300	VGG-16	✓	-	✓	✓	15.0	80.2
ADFPNet300	VGG-16	✓	✓	✓	✓	16.0	81.1

**ADFP** indicates using the proposed ADFP module. **ADEP_L**, indicates adding the ADEP_L module with laige dialtion rate. **AFCB** stands for adaptively feature calibration block. **Time(ms/img)** indicates the time cost for deducing one image in milliseconds.
